# Syndromic Diagnosis of Malaria in Rural Sierra Leone and Proposed Additions to the National Integrated Management of Childhood Illness Guidelines for Fever

**DOI:** 10.4269/ajtmh.2010.09-0188

**Published:** 2010-04

**Authors:** Obinna N. Nnedu, Bryan Rimel, Carey Terry, Heidi Jalloh-Vos, Brima Baryon, Daniel G. Bausch

**Affiliations:** Tulane University Health Sciences Center, New Orleans, Louisiana; Medical Research Centre, Bo, Sierra Leone; Ministry of Health and Sanitation, Government of Sierra Leone, Freetown, Sierra Leone

## Abstract

Many countries in Africa, including Sierra Leone, have adopted artemisinin-based combination therapy as first-line therapy for treatment of patients with malaria. Because laboratory testing is often unavailable in rural areas, the cost-benefit and viability of this approach may depend on accurately diagnosing malaria by using clinical criteria. We assessed the accuracy of syndromic diagnosis for malaria in three peripheral health units in rural Sierra Leone and determined factors that were associated with an accurate malaria diagnosis. Of 175 children diagnosed with malaria on syndromic grounds, 143 (82%) were confirmed by the Paracheck-Pf test. In a multivariate analysis, splenomegaly (*P* = 0.04) was the only clinical sign significantly associated with laboratory-confirmed malaria, and sleeping under a bed net was protective (*P* = 0.05). Our findings show that clinical malaria is diagnosed relatively accurately in rural Sierra Leone. Incorporating bed net use and splenomegaly into the national Integrated Management of Childhood Illness guidelines for evaluation of fever may further enhance diagnostic accuracy for malaria.

## Introduction

Malaria kills approximately one million persons each year, and the major burden of morbidity and mortality is in children less than five years of age, especially in sub-Saharan Africa.^1^ *Plasmodium falciparum* is the main cause of malaria. The disease has the most profound economic impact in developing countries, where it is estimated that 12 billion dollars per year are lost in cost of care and reduced productivity.^2^

Chloroquine and sulfadoxine-pyrimethamine have been the traditional drugs used for treatment of patients with malaria. However, increasing resistance of *P. falciparum* in many countries has prompted calls for changes to this approach. Current recommendations from the World Health Organization (WHO) are for artemisinin-based combination therapy (ACT) as the first-line regimen for patients with uncomplicated malaria.^3^ However, ACT is significantly more expensive than the older anti-malarial drugs, costing up to 10 times the price of the chloroquine.^4^

The Global Fund to Fight AIDS, Tuberculosis, and Malaria has pledged to assist poor countries with the cost of ACT. Nevertheless, resources are inevitably limited. The true cost-benefit, and thus viability of the ACT approach, will hinge partly on the ability to accurately diagnose malaria. Frequent false-positive results would result in unnecessary administration of the more expensive ACT, and perhaps undue adverse effects, and false-negative results would result in delayed treatment, driving up costs and increasing morbidity and mortality.

Laboratory diagnosis of malaria is most frequently made by microscopy, but the sensitivity and specificity of this modality varies greatly with the experience and skill of the microscopist.^5^ Furthermore, microscopes are beyond the financial means of many healthcare facilities, especially in rural areas of sub-Saharan Africa. There are commercially available rapid tests for malaria that are sensitive, specific, and easy to use, but their expense again precludes widespread use.^6^ Most rural health centers therefore rely on syndromic diagnosis. However, the typically non-specific presenting signs and symptoms of malaria make this difficult.^7^

Various algorithms have been developed to facilitate syndromic diagnosis of malaria by healthcare workers in resource-poor settings. The WHO program for Integrated Management of Childhood Illness (IMCI) provides guidelines on how children in resource limited settings should be evaluated and treated.^8^ The World Health Organization recommends that these guidelines may be adapted to suit local health care systems. According to IMCI guidelines, one of the first steps in evaluating a child with fever is to determine the local risk of malaria. A place is considered high risk for malaria if > 5% of fevers among children is caused by malaria. A child with fever in a high-risk area who does not appear to have measles based on physical examination should be classified as having malaria. These children should receive anti-malarial drugs in addition to any other treatments that are deemed necessary based on results of a physical examination.

Sierra Leone is a country on the west coast of Africa and has six million inhabitants. The 12-year civil war in Sierra Leone, which ended in 2002, decimated the nation's economy and infrastructure, including its public health system. The disease is the number one cause of morbidity and mortality in this country and is responsible for up to 48% of visits to healthcare workers.^9^ The high prevalence of chloroquine resistance in Sierra Leone (up to 79% of malaria cases) has led the country to officially adopt ACT as the treatment of choice in accordance with WHO guidelines, but the financial implications of this policy and the burden imposed on the country's recovering public health system have not been fully explored.^9^–^11^ Recognizing the key role that accurate diagnosis plays in determining the cost-benefit of ACT as a first-line therapy for malaria, we conducted a pilot study to assess the accuracy of syndromic diagnosis for malaria in children made by healthcare workers in rural Sierra Leone and to determine what additional factors could be incorporated into the IMCI guidelines to improve malaria diagnosis. Sierra Leone is classified as a high-risk malaria area, which means that children less than five years of age who have fever in the absence of any signs of measles are treated with anti-malarial drugs according to IMCI guidelines. To date, there are no country-specific modifications of the IMCI guidelines for Sierra Leone.^8^

## Methods

### Study site and population.

The research protocol was reviewed and approved by the Ethics Committee of the Sierra Leone Ministry of Health and Sanitation, the Institutional Review Board of Tulane University, and the District Medical Officer in Bo District. The study was conducted in the Bo District of Sierra Leone, where the Medical Research Center is located and has extensive experience. Three rural healthcare centers in Bo (Dambala, Njala Komboya, and Baomahun) known as peripheral health units (PHUs) were selected for study based on accessibility and adequate numbers of patients. Each of these PHUs is headed by a community health officer (CHO) trained in the delivery of basic primary healthcare. In 2005, CHOs typically made a diagnosis of malaria based on the presenting signs and symptoms (i.e., syndromic diagnosis) because microscopy or rapid tests were not available in the PHUs (although their availability has recently been increasing). All children two months to five years of age who came to one of these three PHUs for medical attention in July and August (rainy season) 2005 were eligible for enrollment in the study.

### Questionnaire and laboratory analysis.

After seeing each eligible child, the CHO was requested to complete a brief questionnaire for demographic information on the child, presenting signs and symptoms, use of bed nets, and the diagnosis the CHO ultimately made. The IMCI weight-for-age chart was used; Z scores < −2 indicating low weight and scores < −3 indicating very low weight.^8^ The CHOs were blinded to the purpose of the study to prevent influencing their diagnosis or management of the patients. After the questionnaire was completed, it was reviewed independently by a member of the research team. If a diagnosis of malaria was made by the CHO, informed consent was obtained from the parent or guardian and the child was enrolled in the study. Blood was obtained and tested for *P. falciparum* by using the Paracheck-Pf rapid test (Orchid Biomedical Systems, Goa, India), which has been reported to be 97% sensitive and 88% specific for this parasite.^12^ Children who did not receive a clinical diagnosis of malaria from the CHO were not enrolled.

### Data collection and analysis.

Data on the questionnaire were entered into an Excel spreadsheet (Microsoft, Redmond, WA) and imported into the SAS 9.1 software package (SAS Institute Inc., Cary, NC) for analysis. Two-by-two contingency tables were used to generate crude estimates of association between the factors noted in the questionnaire and the results of the Paracheck test, which was considered the gold standard of malaria diagnosis for the study. Factors with a *P* values ≤ 0.4 level were further explored by using a multivariable logistic regression model. All tests of hypothesis were two-tailed with a type 1 error rate set at 5%.

## Results

One hundred seventy-five children were considered to have malaria by the CHOs at the three PHUs based on the syndromic diagnosis and were enrolled in the study: 63 from Dambala, 44 from Njala Komboya, and 68 from Baomahun (Table 1). The positive predictive value (PPR) of syndromic diagnosis of malaria was 82%. A significantly lower percentage of the syndromic malaria cases were positive by the Paracheck test in Baomahun than in Dambala (*P* = 0.006). Otherwise, there were no significant differences between the PHUs with regard to any demographic variable or Paracheck test result (Table 1). Therefore, aggregated data from all three PHUs are presented.

The results of univariate analysis of the relationship of various demographic and behavioral factors and clinical signs and symptoms with a laboratory-confirmed diagnosis of malaria are shown in Table 2. Palmar pallor (*P* = 0.01) and splenomegaly (*P* = 0.001) were significantly associated with a diagnosis of malaria. Sleeping under a bed net was of borderline statistical significance (*P* = 0.07).

The results of the multivariate analysis are shown in Table 3. After controlling for other variables, children with splenomegaly were three times more likely to have malaria (*P* = 0.04), and those who reported sleeping under bed nets were 70% less likely (*P* = 0.05). Malaria was 13 times more likely in children with fever, but this relationship was not statistically significant (*P* = 0.09).

## Discussion

We assessed the quality of syndromic malaria diagnosis in rural Sierra Leone and identified factors that may enhance its accuracy. The CHOs in the study provided a diagnosis of malaria with a reasonably high PPV of 82%. This finding suggests that assuming that the drugs are accessible, most children who come to a PHU and are considered to have malaria on syndromic grounds would receive ACT, which is favorable in terms of limiting morbidity and mortality. There was significant variation (72–92%) by PHU in the accuracy of the malaria diagnosis made, which could indicate varied clinical skills of the respective CHOs or different baseline incidences of malaria in the catchment areas of the PHUs because PPV is influenced by the baseline frequency of the disease of interest.

We can only speculate as to what other clinical observations were deemed important by the CHOs in assigning a diagnosis of malaria, but it is entirely possible that splenomegaly, a frequent manifestation that was significantly associated with the disease in our study, was one factor that was considered. Interestingly, our data suggest that consideration of the background risk of malaria transmission, in addition to direct clinical observations, can help in the diagnosis because the use of bed nets was shown to be protective.^13^,^14^ This finding also highlights the need for continued distribution and use of bed nets. Despite their demonstrated protective effect, they were used by only 12% of the children we surveyed.

The major shortcoming of our study is that logistical limitations prevented us from including children who did not receive a syndromic diagnosis of malaria. Thus, we cannot calculate the sensitivity, specificity, or negative predictive values of the CHOs diagnoses. A high PPV and sensitivity with a syndromic diagnosis approach could easily be achieved by assuming that most febrile illnesses are malaria, at the cost of a poor specificity and negative predictive value. Although the assumption that fever equals malaria might be considered reasonable in countries such as Sierra Leone, where malaria is endemic, recent findings suggest that malaria may be over-diagnosed even in areas of frequent transmission.^15^ Furthermore, although this approach might be favorable in terms of limiting morbidity and mortality because all children with malaria would be treated, it would be expensive and perhaps unsustainable because ACT would be given to many children who, in fact, would not have the disease. The assumption of malaria would also likely result in delayed recognition and treatment of other diseases. Lastly, the overly liberal use of ACT might promote the development of artemisinin resistance. Drug failure of *P. falciparum* to artemisinin compounds has already been reported in Sierra Leone.^16^

Other limitations of our study include the relatively small sample size and non-random selection of the PHUs, which dictate caution in generalizing our findings to other areas of Sierra Leone or other countries and our inability to determine precisely which factors were used by the CHOs to make a diagnosis of malaria. We cannot conclude that the experience and skills of the CHOs in this study are the normal situation in Sierra Leone, although there is no particular reason to believe that they are not representative. Lastly, because the Paracheck test detects only *P. falciparum*, it is possible that cases of malaria caused by *P. malariae* or *P. ovale* were missed, although these parasites account for a small proportion of malaria in Sierra Leone.^17^,^18^

In summary, data from this pilot study show that the PPV of syndromic diagnosis for malaria in Sierra Leone is reasonably high, and it might be further improved by including questions about bed net use and emphasizing the importance of a thorough evaluation for splenomegaly in the evaluation of fever among children. Consideration should be given to including these measures in the Sierra Leone National IMCI guidelines. Future studies should include children thought to have malaria and other diagnoses to determine the sensitivity, specificity, and negative predictive values for syndromic malaria diagnosis. Furthermore, a larger number of PHUs and CHOs ideally distributed around the country should be studied to better assess inter-observer and inter-regional variability in Sierra Leone.

## Figures and Tables

**Table 1:** Demographic information and Paracheck-Pf results on 175 children suspected to have malaria based on syndromic diagnosis, Sierra Leone

Variable	No. (%) enrolled (n = 175)	Paracheck test result	
		No. (%) positive (n = 143)	No. (%) negative (n = 32)
Peripheral health unit			
Dambala*	63 (36)	58 (92)	5 (8)
Njala Komboya	44 (25)	36 (82)	8 (16)
Baomahun*	68 (39)	49 (72)	19 (28)
All	175 (100)	143 (82)	32 (18)
Age, months			
< 12	88 (50)	74 (84)	14 (16)
≥ 12	87 (50)	69 (79)	18 (21)
Sex†			
Male	82 (48)	70 (85)	12 (15)
Female	89 (52)	71 (80)	18 (20)

* *P* = 0.006 for Dambala versus Boamahun.

† Sex data were missing for four patients.

**Table 2:** Univariate analysis of factors associated with malaria diagnosis as determined by the Paracheck-Pf rapid test, Sierra Leone

Variable	Paracheck test, no. (%) positive (n = 143)	Paracheck test, no. (%) negative (n = 32)	Crude odds ratio (95% confidence interval)	*P*
Age, months				
< 12	74 (84)	14 (16)	1.0	0.4
≥ 12	69 (79)	18 (21)	0.7 (0.3–1.6)	
Sex*				
Female	70 (85)	12 (15)	1.0	0.4
Male	71 (80)	18 (20)	0.7 (0.3–1.5)	
Weight-for-age				
Normal	79 (57)	17 (57)	1.0	0.8
Very low	34 (24)	6 (20)	1.2 (0.4–3.4)	
Sleeps under bed net				
No	122 (88)	22 (76)	1.0	0.07
Yes	16 (12)	7 (24)	0.4 (0.2–1.1)	
Fever†				
No	1 (1)	1 (3)	1.0	0.2
Yes	142 (99)	31 (97)	4.6 (0.3–75.2)	
Palmar pallor				
No	96 (68)	28 (90)	1.0	0.01
Yes	46 (32)	3 (10)	4.5 (1.3–13.5)	
Splenomegaly				
No	68 (50)	23 (82)	1.0	0.001
Yes	68 (50)	5 (18)	4.6 (1.6–12.8)	
Lethargy				
No	127 (89)	30 (97)	1.0	0.2
Yes	16 (11)	1 (3)	3.8 (0.5–29.6)	
Cough				
No	27 (19)	3 (9)	1.0	0.2
Yes	116 (81)	29 (91)	0.4 (0.1–1.6)	
Vomiting				
No	80 (56)	18 (58)	1.0	0.9
Yes	62 (44)	13 (42)	1.1 (0.5–2.4)	
Diarrhea				
No	130 (91)	29 (94)	1.0	0.6
Yes	13 (9)	2 (6)	1.4 (0.3–6.8)	
Poor feeding				
No	90 (63)	17 (55)	1.0	0.4
Yes	53 (37)	14 (45)	0.7 (0.3–1.6)	

* Sex data were missing for four patients.

† Refers to a subjective feeling of fever as reported by the patients and/or the community health officer's impression through touching and observing the patient. Temperatures were not consistently taken at all peripheral health units.

**Table 3:** Multivariate analysis of factors associated with malaria diagnosis as determined by the Paracheck-Pf rapid test, Sierra Leone

Variable	Odds ratio (95% confidence interval)	*P*
Age, months		
< 12	1.0	0.55
≥ 12	0.7 (0.3–2.0)	
Sleeps under bed net		
No	1.0	0.05
Yes	0.3 (0.1–0.98)	
Fever*		
No	1.0	0.09
Yes	13.3 (0.7–263.4)	
Palmar pallor		
No	1.0	0.16
Yes	2.8 (0.6–12.1)	
Splenomegaly		
No	1.0	0.04
Yes	3.2 (1.02–10.1)	
Lethargy		
No	1.0	0.55
Yes	2.0 (0.2–17.2)	
Cough		
No	1.0	0.27
Yes	0.4 (0.1–1.8)	

* Refers to a subjective feeling of fever as reported by the patients. Temperatures were not consistently taken at all peripheral health units.
